# Recovery after aerobic exercise is manipulated by tempo change in a rhythmic sound pattern, as indicated by autonomic reaction on heart functioning

**DOI:** 10.3389/fnhum.2014.00738

**Published:** 2014-09-19

**Authors:** John Wallert, Guy Madison

**Affiliations:** Department of Psychology, Umeå UniversityUmeå, Sweden

**Keywords:** recovery, exercise, anisochronous, sound pattern, autonomic nervous system, heart rate variability, rhythmic entrainment

## Abstract

Physical prowess is associated with rapid recovery from exhaustion. Here we examined whether recovery from aerobic exercise could be manipulated with a rhythmic sound pattern that either decreased or increased in tempo. Six men and six women exercised repeatedly for six minutes on a cycle ergometer at 60 percent of their individual maximal oxygen consumption, and then relaxed for six minutes while listening to one of two sound pattern conditions, which seemed to infinitely either decrease or increase in tempo, during which heart and breathing activity was measured. Participants exhibited more high-frequent heart rate variability when listening to decreasing tempo than when listening to increasing tempo, accompanied by a non-significant trend towards lower heart rate. The results show that neuropsychological entrainment to a sound pattern may directly affect the autonomic nervous system, which in turn may facilitate physiological recovery after exercise. Applications using rhythmic entrainment to aid physical recovery are discussed.

## Introduction

The ability to recover quickly after exercise is desirable because it facilitates subsequent high performance. Athletes display such accelerated recovery while both hampered performance and decelerated recovery is observed in physically impaired (Imai et al., 1994; Seiler et al., 2007; Kontsas et al., 2013), malnourished (Bollaert et al., 1992; Birmingham and Tan, 2003), overtrained (Budgett, 1998), and sleep deprived (Samuels, 2009) individuals. In addition to boosting performance, both endurance and strength training seems to accelerate recovery after exercise (Tomlin and Wenger, 2001; Heffernan et al., 2007; Otsuki et al., 2007). Moreover, subsequent detraining has been found to precede regression of such recovery-acceleration as subjects return to their former lower level of fitness (Sinacore et al., 1993; Heffernan et al., 2007). These converging data suggest a close interplay where the quality of performance is dependent upon the quality of recovery. Hence, it is worthwhile to explore novel ways to manipulate performance indirectly through recovery.

Different techniques are used to recover faster, including stretching, light exercise and rest (Williams and Harris, 2006; Mika et al., 2007). Several of these techniques include periodic elements, for example breathing rhythms and mantras in various meditational practices (Halsband et al., 2009; Yu et al., 2011), as well as listening to rhythmic music (Karageorghis and Terry, 2008). The extensive use of music for this purpose seems to indicate that it has particularly powerful effects, but data are rather inconclusive. It can be concluded that music is used extensively as an intervention, and found to reduce pain perception (Barker, 1991; Cepeda et al., 2006; Pothoulaki et al., 2008), induce relaxation (Barker, 1991; Smith and Joyce, 2004; de Niet et al., 2009), reduce heart rate (Barker, 1991) and reduce stress (Guzzetta, 1989) - variables often measured when assessing recovery. On the other hand, studies involving effects of music have repeatedly reported contradicting or null results (Good, 1995; Bartlett, 1996; Lazic and Ogilvie, 2007). See Bartlett (1996) for a comprehensive review of physiological responses to music, and Vasionytė and Madison (2013) for a review of music’s effect with dementia patients. In light of these inconclusive results it remains unclear why the use of music in conjunction with exercise continues to increase (Yamashita et al., 2006), and why we subjectively attest to its effects on our capacity to perform and recover (Priest and Karageorghis, 2008). It has been proposed that there are true effects, but that the difficulty to disentangle components of the musical stimulus leads to inconsistent results (Iwanaga and Moroki, 1999; Ellis, 2009). Music constitutes a complex stimulus that involves several more or less independent aspects or components, such as rhythm, melody, harmony, lyrics, etc. Therefore, the “active component” in music used in intervention studies may be one or the other, or several operating in complex interactions with each other. Also, the influence of music might lie more in recovery and relaxation between bouts of performance. The available empirical data is insufficient to delineate the extent of these possibilities, which highlights the need for tighter experimental control, in particular regarding music stimulus conditions.

One can avoid the problem with stimulus complexity by focusing on rhythmic entrainment without most other features characteristic of music (Ellis, 2009; Eliakim et al., 2013). This focus is motivated by the fact that almost all music used for recovery and performance enhancement is rhythmic and thus enables entrainment. This suggests that entrainment may be an essential component of the music-recovery relation worthy of study in its own right. Entrainment is defined as the synchronizing process that occurs when independent rhythmic systems interact (Clayton, 2012). Synchronization among individuals would not be possible without the ability to entrain (Phillips-Silver et al., 2010; Phillips-Silver and Keller, 2012). It has been proposed that human entrainment is made possible by our capacity to (a) perceive stimuli as rhythmic; (b) produce rhythmic stimuli; and (c) combine them both utilizing sensory feedback (Phillips-Silver et al., 2010). These capacities make it possible to proactively predict when the next rhythmic event will occur so that motor behavior can be produced in precise temporal alignment with such events, something that would be impossible if we merely reacted on each event after it had occurred. Hence, this predictive timing is regarded as a prerequisite for synchronizing movements in group settings (Madison, 2009; Merker et al., 2009). Moreover, focusing on entrainment allows for applying highly controlled stimuli with deterministic properties, making the specific musical content itself irrelevant. In support of this approach, we note that several studies have found relationships between exercise intensity and the preferred tempo of concurrent music, as reviewed in Karageorghis and Terry (2008), and also effects of rhythm *per se* on recovery (Eliakim et al., 2013).

Entrainment is also a robust physiological phenomenon at the micro-level of electrochemical activity in neuronal groupings in various parts of the mammalian brain (Ward, 2003; Gomez-Ramirez et al., 2011; Lavallee et al., 2011; Heinrichs-Graham and Wilson, 2012; Henry and Obleser, 2012). Entrainment at the level of neuronal oscillations has been directly or indirectly linked to how entrainment influences human behavior at the macro-level, such as generating faster reaction speed (Stefanics et al., 2010), enhanced attention (Jones et al., 2002; Henry and Obleser, 2012), less depression (Cantor and Stevens, 2009), and improved intentional motor behavior (Thaut et al., 1997; Sommer and Rönnqvist, 2009; Johansson et al., 2012). These well-established observations suggest three important things. First, human entrainment is a reliable biological response that occurs across the whole range from basic nerve cell activity to overt behavior, both voluntarily (e.g., conscious motor coordination) and involuntarily (e.g., unconscious neuronal oscillations). Second, with the use of entrainable rhythmic stimuli it should within limits be possible not only to entice a biologically measurable response, but also manipulate it by means of the stimulus-specific properties. Third, this response is likely to involve the general nervous system function and therefore be detectable in at least some select sub-parts, such as the autonomic nervous system (ANS). It should be noted that whereas entrainment refers to the synchronization of a neural predictive process with a stimulus, overt entrainment is neither performed nor measured in the present study. This is necessary because participant movement would otherwise constitute a confounding variable of direct influence on heart functioning (e.g., Lunt et al., 2011).

In summary, rhythmic techniques involving entrainment are used frequently to boost recovery, possibly because the ANS can be down-regulated by entraining to rhythmic stimuli with an appropriate tempo, for example one that feels relaxing or one that is slightly slower than optimal for the preceding physical exercise. We propose that a stimulus with infinitely decreasing tempo should even more efficiently enhance relaxation. Moreover, such a stimulus would be more general because it can be the same for all participants and situations, as it is not fixed to any one tempo. The effectiveness of such a stimulus is plausible because the rate of a subjective pulse signifies the rate of bodily movement, which in turn is related to energy expenditure, and so forth. While athletes choose their music and its tempo carefully for each need, e.g., a fast tempo for high speed treadmill walking or a slow tempo for relaxation (Karageorghis and Terry, 2008), we generalize this to increasing and decreasing tempo, respectively. In this way we also avoid the issue of matching a particular tempo, because the generalized stimulus entrains either to the slowest or the fastest tempo each individual is inclined to entrain to (Madison, 2009).

The stimulus that is applied for this purpose is called Multiple Level Pattern (MLP), which refers to multiple temporal levels as found in music and reflected by the binary division of time used in music notation. The algorithm underlying the sound pattern is described in detail in Madison (2009). The MLP is amenable to a series of manipulations that create an illusion of a continuously increasing or decreasing tempo, based on certain properties of the human perception of temporal regularity. For the present purposes, we devise a slow linear change in the pattern’s inter-event-intervals, well within our limits to use for predictive timing. When this interval has doubled (in the case of tempo decrease) or halved (in the case of tempo increase) the pattern sequence is repeated from the beginning, but with the loudness of the various rhythmical levels arranged such that the repetition boundary is obscured. Under these conditions, the individuals’ internal representation of the pulse is so salient that it “tags on” to a slower level in the pattern (in the case of a tempo decrease) as previously documented (Madison, 2009). Another important point is that this kind of stimulus is uniform, apart from the slow tempo change, which renders any section of the stimulus identical in its central perceptual function (increase/decrease) and also highly similar in its surface properties. Hence, the risk of confounding variables or unsystematic variability due to stimulus properties is greatly reduced when employing the MLP. To summarize, the present stimulus is purely rhythmic and thus avoids possible confounds that cannot fully be controlled in musical or music-like stimuli because of the sheer number of stimulus properties, and, it is also perfectly reversible. Together, these two properties provide a very high level of experimental control compared to previous studies using real music or music-like stimuli. To the best of our knowledge, this is the first study to use the MLP to investigate if entrainment rate may affect physical recovery.

In addition to heart rate (HR) *per se* it was of particular interest to detect possible inhibitory or excitatory regulation of bodily activity mediated by the ANS. Effects of stimuli on the workings of this system can be indirectly studied through heart rate variability (HRV). Heart rate variability can be partitioned in different frequency components similar to how the electroencephalogram power is divided into alpha, beta, etc. bands. It was originally suggested that a low frequency (LF) and a high frequency (HF) HRV component indicate variation in sympathetic and parasympathetic nervous system activity (Sayers, 1973; Akselrod et al., 1981). Aggregate data establish that HF power reflects parasympathetic nervous system (PNS) activity, while LF power reflects both sympathetic nervous system (SNS) activity and PNS activity (Camm et al., 1996). It has been suggested that increased LF components in HRV reflects more autonomic sympathetic activity of the heart, and that, conversely, increased HF activity reflects inhibition of sympathetic activity (Pagani et al., 1997). However, increased background SNS activity was accompanied by increased chronotropic activity in the PNS, suggesting that possible concurrent PNS increase must also be considered when studying bodily up-regulation through the change in heart functioning governed by SNS (Uijtdehaage and Thayer, 2000). Relating HRV to music, it has been found that so-called excitative music produced less HF power, indicating decreased PNS activation (Iwanaga et al., 2005), as would be intuitively expected. Further investigation in the context of rhythm have shown that HRV decreased as a function of faster isochronous rhythm, indicating that faster tempo induced a withdrawal of the steady-state resting PNS activity and subsequent “let-go” effect on released and surging SNS activity (Ellis, 2009). Such relationships have also been labeled as a *tonic inhibition*, exercised by the PNS on heart activity through the vagus nerve (Thayer and Lane, 2000, 2009; Ellis and Thayer, 2010). This tonic inhibition dominates during rest but is gradually lifted as SNS activity increases due to physical activity (Ellis and Thayer, 2010). In conclusion, interacting mechanisms exert cardiovascular control following both PNS and SNS activity, which have to be considered when interpreting HRV indicators (Camm et al., 1996; Uijtdehaage and Thayer, 2000; Weippert et al., 2013). Although some have argued that compound measurements of HRV frequency distribution such as LF/HF ratio, as a proxy for sympathovagal balance, should be abandoned pending further theoretical development, others stress that the usefulness of sympathovagal balance is in relation to what research questions are asked (Perini and Veicsteinas, 2003; Pagani et al., 2012). We argue that it is highly relevant for the present study, since it is related to the intensification-relaxation continuum of bodily activity reflected in sympathovagal balance of the heart (Pagani et al., 1986, 1997, 2012; Camm et al., 1996; Perini and Veicsteinas, 2003; Chuang et al., 2010; Boudreau et al., 2012), and therefore likely to reflect the quality of recovery. Dominant PNS activity reflected in more HF of HRV has also been observed during rest in supine position and at sea level, while LF activity was found to increase when subjects assume sitting posture and when suffering from hypoxia induced by high-altitude. During recovery after exercise, PNS assumes vagal control rapidly during the first minute after exercise while SNS reacts later, slowly reducing its activity during the second to fifth minute (Perini and Veicsteinas, 2003). These conditions fit well with the design of this study to derive LF/HF of HRV during recovery after exercise with the participants’ studied at sea level, relaxing in supine position, with eyes shut while listening to the MLP for 6 min.

We hypothesized that physically active adults would recover faster from aerobic exercise when they heard a repetitive sound pattern that seemed to decrease in tempo, compared to when it seemed to increase in tempo. Faster recovery was defined as faster decrease in mean HR, a smaller LF/HF frequency component ratio in HRV, and faster decrease in mean breathing cycles (BC). It was further hypothesized that listening to decreasing tempo would be associated with a higher level of self-rated calmness and relaxation and higher positive affect when compared to the inverse condition.

## Materials and methods

### Statement

The study was approved by the local ethics review committee (Regionala Etikprövningsnämnden) in Umeå (Dnr 2012-211-31M). The study was conducted in accordance with the Declaration of Helsinki. All participants signed a written informed consent before participating, and each received monetary compensation equivalent to 40 USD, and individual max-test results (as detailed below, worth approximately 120 USD).

### Participants

Twelve participants (six male and six female, *M* = 24 yrs, *SD* = 3.41) were recruited through billboards and various Internet fora linked to Umeå University in Sweden. The following information was given: the purpose of investigating physically active adults’ ability to recover after exercise, with participants required to complete a maximal test on a cycle ergometer (Ramp-test, Test 1) at the University’s Sports Medicine Unit laboratory, and attend six sessions of submaximal tests on cycle ergometer (Submax tests, Test 2–7). During recovery after the submaximal tests, the participants would lie down while listening to different sounds through headphones. Compensation and the applicant procedure was stated explicitly. A telephone interview followed to establish suitability according to in- and exclusion criteria. Inclusion criteria were: 20–35 years old, physically active individuals who exercised regularly, defined as at least 3 × 30 min exercise-periods per week maintained for at least 30 days prior to the first test session. Exclusion criteria were: pregnancy, use of depressants, non-caffeine stimulants, alcohol, ongoing infection, fractures, diagnosed heart disease, anxiety, sleep disturbances, chronic pain or other relevant health complications. Further requirements were: E-mail and telephone access, to document sleep, food intake and deviations from normality in a personal health diary during experimental testing. Food intake and training routines required abstaining from (a) heavy exercise ≤24 h; (b) eating a large meal ≤3 h; and (c) eating a small meal ≤1 h before experimental sessions. To safeguard participants’ health and improve test-retest reliability, it was required that each participant was physically active relative to norms presented in a major review of healthy untrained subjects in the USA, Canada and Europe (Shvartz and Reibold, 1990). Thereafter, participants were scheduled for the six testing sessions that were completed within a period of 3–4 weeks. One participant’s data was excluded due to sleep disturbances. He was allowed to complete the testing and received full compensation.

### General study design

The present study aimed to establish a possible causal relation between direction of tempo change and recovery-time after exercise. All physiological data was therefore collected in an exercise laboratory at Umeå University, assuring that nuisance variables were kept constant. It was likely that physiological differences between participants would obscure systematic experimental effects, and a within-participant design was therefore used. Even when having each participant as its own control, variation in states, such as fatigue, glucose, or mood, might blur induced differences between sessions. Also, because of the repeated measures, significant learning/ordering-effects were possible. To counter this, three sessions per experimental condition were conducted considering that a total of six sessions would be a reasonable compromise in order to avoid attrition and achieve proper counterbalancing. Counterbalancing demanded half of the participants divided into two groups, each starting with one of the two conditions and then alternating conditions until 3 × 2 sessions were completed for each participant. Repeated-measures effects due to potential fatigue were controlled for by the spacing between and the requirement of rest before each session. It was expected that learning/ordering-effects would be negligible with these applied countermeasures. Furthermore, comparable effects across participants were attained by individualized levels of workload determined by individual maximal aerobic capacity (VO_2_max), indicating physical fitness as detailed below. To ensure comparability over all submaximal and maximal test-sessions, participants exercised on the same cycle ergometer (818E, Monark, Vansbro, Sweden). This ergometer adjusts workload to cadence when cycling so if the latter fluctuates, a constant amount of power is still produced. Instructions were to keep the cadence between 60–70 rpm since the adjusting function may become unreliable at extreme values. The cadence range, rather than a specific value was chosen for participant comfort.

#### Procedure and measurements for determining VO_2_max

The Ramp-test is an incremental maximal test that has been used in other studies of fitness in physically active adults (Larsson and Henriksson-Larsén, 2005; Gilenstam et al., 2011). It is also specifically designed for low exercise thresholds seldom measured with maximal tests. Before Ramp-testing, participants answered a standardized questionnaire regarding health status and training frequency, were weighed with a flat scale (Seca M877, GmbH & Co. KG, Hamburg, Germany), fitted with a mouthpiece (7400 Vmask, Hans Rudolph Inc., Kansas, USA), a thoracic pulse-band (RS800CX Wearlink Coded, Polar Electro, Kempele, Finland) and instructed to adjust the seat and handle of the ergometer to their liking. A professional operator guided the participants and supervised all Ramp-testing.

The Ramp-test was executed incrementally on the ergometer until maximal exertion, meaning that cycling was initiated and kept at 60–70 cadence, becoming increasingly difficult as physical resistance elevated. Test completion occurred when pedaling speed dropped below 60 cadence due to exhaustion (judged by the professional operator). Base and step-wise workload increase was 30 watt for females and 40 watt for males, with 3 min for each incremental step. Test completion time varied from 15 to 25 min. The total volume of expired carbon dioxide (VCO_2_) and inspired oxygen (VO_2_) was analyzed at 30 s intervals by a pulmonary measuring system (Oxycon Pro Jaeger, Carefusion, San Diego, USA) connected to the mouthpiece, together with breathing frequency. The gas analysis was calibrated with a three-point procedure in relation to known gases. Data was monitored and analyzed with a PC-computer using designated software (LabManager, version 5.3). Heart rate was registered each minute with a pulse-watch (Sport Tester, Polar Electro, Kempele, Finland) receiving data from the thoracic pulse-band.

Ramp test maximal workload was later used to determine the submaximal workload for the following six submaximal tests. Several common measures were gathered at (i) exhaustion; (ii) specific submaximal thresholds; or (iii) every incremental level of exertion, as appropriate for; VO_2_max, Maximal heart rate per minute (HRmax), Aerobic threshold (Ventilatory), Anaerobic threshold (Wasserman), the first time when the VCO_2_/VO_2_ quotient reaches 1 indicating exclusive glycolytic work in the body (RER 1) and time endured on the highest workload.

#### Procedure for determining VO_2_submax

In order for participants to produce the individually appropriate amount of work they had both to attain maximal stroke volume plateau and avoid reaching the lactate threshold. In other words, we made sure that (a) participants exercised *enough* so that their hearts had reached the maximal stroke volume plateau, defined in previous studies as occurring at 40–50% of individual VO_2_max (Higginbotham et al., 1986; Zhou et al., 2001). Since heart stroke volume reaches a plateau in most subjects, cardiac output is at this plateau solely dependent on HR which then becomes optimal at indicating the amount of physical exertion and recovery. At the same time, we ensured that (b) participants exercised at a *lower* intensity than their lactate threshold, defined in physically active adults as 70–80% of individual VO_2_max (Demello et al., 1987; Åstrand et al., 2003). Reaching the lactate threshold indicates that the body is making a transition from aerobic to anaerobic energy consumption, rapidly switching to use more glucose for metabolism and therefore potentially skewing respiration-, cardiovascular and performance data (Åstrand et al., 2003). Considering both (a) and (b), 60% of each participant’s VO_2_max was chosen as the amount of physical exertion for the submaximal tests. As explained, data from the Ramp-test was then used to determine what amount of work corresponded to 60% of individual VO_2_max. This ranged from 120–180 watt between participants due to varying physical capacity. Recovery was operationalized as the decrease in heart rate after a steady-state submaximal workload for 6 min, as detailed by Åstrand and others (Åstrand et al., 2003; Andersson, 2011). The submaximal test is validated and produces reliable repeat-measurements of steady-state activity (Åstrand et al., 2003; Andersson, 2011), a requirement for comparability across the six experimental sessions. The ideal use of this test is with physically active subjects free from infections and of normal weight (Andersson, 2011), criteria that were met with the exception of two minor colds.

#### Procedure and measurements for the experimental sessions

After completing the Ramp-test, participants were scheduled for six experimental sessions. To avoid performance-effects of variations in the awake-sleep cycle and fatigue, each participant’s total number of sessions were scheduled at the same part of the day with no more than 1 h difference and at least 24 h of spacing between consecutive sessions. To avoid individual fitness-status becoming obsolete, each participant completed all testing within 3 weeks after Ramp-testing. They were also instructed to maintain usual training-frequency, limiting both exercise and food-intake as detailed above. If participants reported any deviations from instructions, and if judged to affect performance or participants’ health, the session was cancelled and re-scheduled.

Detailed information was given on the first session. The amount of power, clothing (shorts/athletic pants/jeans), grip of the handlebar (overgrip/undergrip), type of saddle, and the height and depth of the saddle was kept constant over sessions for each participant. Sessions began with the participant seated on a bed answering a set of verbally administered standard questions targeting current health status, food and substance intake, unusual physical pain, exercise activity, and sleep activity. The diary was then checked for further deviations. Blood pressure (BP) and HR were then measured. If it was the first session, the participant was asked to adjust the cycle ergometer to their liking, and the adjustment was documented. The participant was then outfitted with the respiratory transducer, ECG electrodes and the IR-sensor, and asked to mount the ergometer. Communicating any pain or discomfort during testing was encouraged. At this point the experimenter was informed about the behavior required immediately after the cycling was finished and during recovery phase. Instruction was then given to start pedaling and keep the cadence at 60–70, joint-monitored via the ergometer display. The experimenter set a timer to 6 min, marking the start and duration of the submaximal test. During the submaximal test, data recording and patient behavior was corrected if needed by the experimenter such as adjusting the electronic equipment, reminding the participant to maintain cadence, not to speak or change type of ergometer handgrip. When 15 s of the test remained, the participant was notified. Directly after completing the submaximal test, instruction was given to (1) stop pedaling; (2) dismount; (3) lay down in supine position on the bed; (4) relax the muscles, and arms along the body side; (5) report any discomfort or otherwise signal thumbs-up when the experimenter placed the headphones on the participant; and (6) keep eyes closed and lay still until the experimenter removed the headphones. After completing points (1)–(6), the sound pattern was administered simultaneously with initiating the recording of physiological data. This marked the start of the recovery-phase. The experimenter then moved a designated few feet away to ensure experimenter blindness of condition and keeping relatively constant the effect of experimenter presence. This continued until the stimulus pre-programmed 6 min ended. The headphones were removed and the participant asked to sit up and answer the questionnaires (PANAS and DCR, detailed below). Thereafter the participant was stripped of the electronic equipment and the session finished.

##### Sound pattern

**Figure 1:**
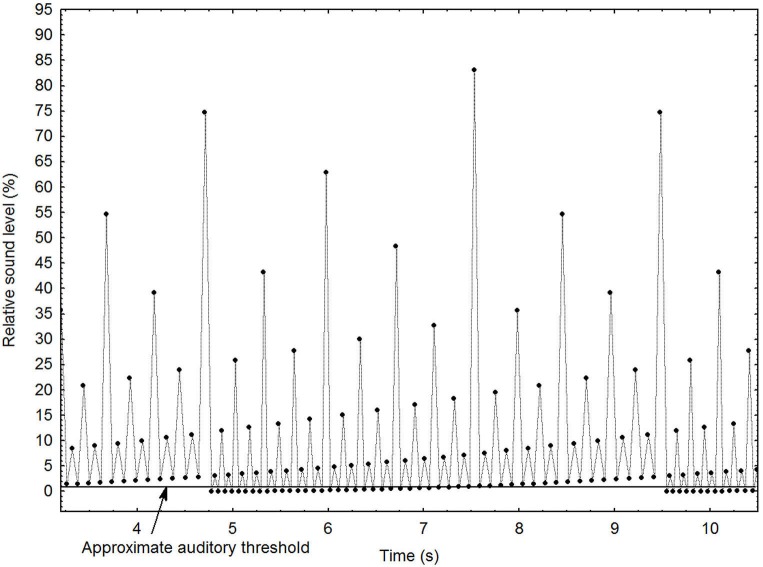
**Graphical depiction of the main principles for the stimulus pattern with decreasing tempo**. Each dot represents a sound event. See text for further details.

For this particular study, the MLP was subjected to a series of manipulations that create an illusion of a continuously increasing or decreasing tempo. These are described in detail elsewhere (Madison, 2009). Figure 1 depicts the general principles of the stimulus, using a shorter pattern for clarity (96 events) than the one employed in the present study (1536 events). It shows the local interval increasing gradually until it has become double that of the beginning of the pattern, which occurs at ~4.75 and ~9.5 s, at which point it is immediately and seamlessly brought back to the initial interval. The ordinate depicts the relative loudness of each sound event, and shows that loudness is assigned according to a hierarchical binary division, which creates the percept of a pulse with many alternative auditory levels. As the most extreme levels are partially obscured by the listener’s auditory threshold, the salient levels seem to be constantly present, and the boundary when the pattern is repeated is unnoticeable. A systematic manipulation of the relative loudness among all levels makes the loudness of one level equal to the loudness of the next higher or lower level, which completes the illusion that the tempo changes infinitely, although it never reaches extremely fast or slow manifest levels. This is because under these circumstances the level in the pattern that the entraining subject chooses to lag on to is entirely subjective. When the entrained tempo becomes uncomfortable, it switches automatically to one or two levels higher or lower, depending on the direction of the change, clearly demonstrated elsewhere (Madison and Merker, 2005). For administration of the sound patterns and blocking of other sounds, a pair of combined hearing protectors/headphones (PELTOR HTP79A, 3M Company, Saint Paul, Minnesota) were used. Sound patterns were played at approximately 72 dBA SPL.

##### Heart rate, breathing cycles, and blood pressure

Heart rate was measured with three electrodes attached at standard positions on the chest (RA; below the right clavicle, LA; below the left clavicle, and LL; below the left pectoral muscle) using surgical tape and designated paste after cleaning with rubbing alcohol and skin-abrasive. A SIRECUST 311S ECG-monitor (Siemens, Munich, Germany) detected R-R intervals and produced a pulse output for each heart beat that was recorded by the computer producing the stimulus signal. A back-up was also recorded using an infrared (IR) pulse sensor attached to participant’s right ear lobe (MLT1020EC, AD Instruments, Dunedin, New Zeeland). The pulmonary cycle rate was measured indirectly with a piezo-electric respiration transducer that registers expansion/contraction of the thorax (model 1132 Pneumotrace II, UFI, Morro Bay, California). Blood pressure comprises a possible confound for HR (Loft et al., 2007; Conley and Lehman, 2012), and was therefore assessed when initiating each submaximal session on the upper left arm with a HR-monitor (Omron M2, Omron Healthcare Europe, Hoofddorp, the Netherlands).

##### Psychological measures

The positive and negative affect schedule (PANAS) is a 20-item questionnaire, subdivided in two 10-item subscales that measure independent positive affect (PA) and negative affect (NA). Items are answered by rating (from 1–5) the magnitude of affective states experienced in the moment, such as anger, excitement and interest. The PANAS was chosen because it has shown high internal consistency, test-retest reliability and stability over at least a 2-month period (Watson et al., 1988; Crawford and Henry, 2004) and has been used with a Swedish sample before (Garcia and Moradi, 2012). The positive and negative affect schedule aim to partially reflect stable, probably genetic emotion-temperamental dispositions (Lykken and Tellegen, 1996) and probably relates to how biological emotionality differs in individuals. Any systematic difference in PANAS subscales due to exposure to decreasing or increasing tempo would indicate a direct effect of entrainment on individual affective mood-states reflecting subjective well-being or lack thereof. The degree of calmness and relaxation (DCR) consisted of one item with a nine point Likert-type scale where participants assessed their degree of calmness and relaxation ranging from (1) “Very little or not at all” to (9) “Very much”.

##### Data registration

The signal from the ECG-monitor was directed through a Alesis DM-5 MIDI trigger (Cumberland, Rhode Island) and a Roland MPU-401 (Hamamatsu, Japan) MIDI interface into a computer (OptiPlex GX1, Pentium II, Dell). Custom software recorded the inter-onset-intervals (IOIs) between heart beats and produced the stimulus events that were converted into sounds by the DM5 unit’s drum sound module. IR-sensor HR, piezo-electrically transduced BC, and the stimulus signal were registered by a Powerlab 8/30 electrophysiology amplifier (AD Instruments) and recorded in another computer (Pentium 2, Hewlett-Packard, Palo Alto, California) using the provided software Chart 5 (AD Instruments). Demographic- and descriptive data was registered in Statistica (Statsoft Inc, Tulsa, Oklahoma).

### HRV analysis

The relative power of frequency components in the ECG-data over the eight consecutive time-series-intervals was assessed with Detrended Fluctuation Analysis (DFA; Peng et al., 1994). Detrended Fluctuation Analysis is a power spectral method appropriate for analyzing 1/f noise, in which there is a systematic relation between power and frequency across the frequency spectrum (Peng et al., 1995; Madison, 2004). Biological phenomena that produce 1/f noise include HR and neuronal activity. 1/f noise has regular properties but the statistical structure violates assumptions of stationarity and random (uncorrelated) errors, and can therefore not be meaningfully analyzed with linear statistics. A method such as DFA is required for the present study because interest lies in changes within less than a minute and to detect these changes during a few minutes of physical recovery. Fourier analysis, which is the standard method for detecting HF and LF components, requires a minimum of a few hundred data points, which corresponds to at least 5 min of data recording. To detect changes within fractions of this time there is a need to use a fractal method that can work with less than 100 data points, and DFA has been found to work for series as short as 64 data points (Madison, 2006). Because physiological participant recovery was expected to be highest at the start of recovery and thereafter diminish, and the usage of DFA was planned for smaller windows of data points, the total time of 6 min spent during recovery was divided into time segments of 2 (180 s), 4 (90 s), 8 (45 s) and 16 (22,5 s). Arithmetic means and standard deviations for each of these segmentations were used in statistical treatment of data.

### Additional analysis

The present analyses were based on arithmetic means, standard deviations, and percentages unless explained otherwise. Descriptive data is presented in Table 1. Repeated measures ANOVAs were applied for determining multivariate main and interaction effects. The alpha level was set to 0.05 (two tailed). Data was analyzed in Statistica (Statsoft Inc.).

**Table 1:** **Individual data for exertion and exercise parameters**.

**Male**	**A**	**B**	**C**	**D**	**E**	**F**
HRmax* (f/min)	188	188	193	189	x	189
VO_2_max** (l/min)	4.4	4.0	4.6	4.0	x	4.2
VO_2_submax*** (l/min)	2.64	2.4	2.76	2.4	x	2.52
Wsubmax**** (watt)	160	150	160	170	x	160
**Female**	**G**	**H**	**I**	**J**	**K**	**L**
HRmax* (f/min)	192	176	187	170	190	190
VO_2_max** (l/min)	3.3	3.5	3.3	3.2	3.2	3.2
VO_2_submax*** (l/min)	1.98	2.1	1.98	1.92	1.92	1.92
Wsubmax**** (watt)	120	180	120	120	120	120

From top to bottom for each sex beginning with participant code letter (A-L). * = Maximal heart rate from the Ramp-test measured as frequency per minute. ** = Maximal oxygen uptake from the Ramp-test measured as ventilatory oxygen consumption in liters per minute. *** = Calculated estimates of 60% of each individual maximal oxygen uptake and, **** = corresponding workload in watt used for the submaximal tests. Male E represents excluded data.

## Results

No systolic or diastolic BP diverged more than two standard deviations from the within-participant arithmetic mean, which was our outlier criterion. Blood pressure was thus an unlikely confounding variable and excluded from further analysis.

Mean VO_2_max was 3.28 l/min (*SD* = 0.117) for the females and 4.24 l/min (*SD* = 0.261) for the males. This is substantially above the selected norm of healthy, untrained subjects in Canada, Europe, and USA (Shvartz and Reibold, 1990). In this review, VO_2_max for the relevant age range was 2.2–1.8 l/min for females and 3.4–3.2 l/min for males. Relative differences to norms were 129% for males and 164% for females. None of the participants were lower than 121% of these norms (participant B and D). This confirms that our participants were physically active (see Table 1 for individual details).

Breathing cycles (BC) were determined from the waveform recorded in the Chart software, for each of the eight 45 s recovery time segments. A three-way (2 direction of tempo change × 8 recovery time segment × 3 session) repeated measures ANOVA with BC as dependent variable showed no main effect of direction of tempo change (*F*_1,48_ = 2.63, *p* = 0.1325; 16.5 for increasing tempo and 16.0 for decreasing tempo) or sessions (*F*_2,48_ = 0.82, *p* = 0.45), or any significant interaction effects. The effect of recovery time segment was statistically significant (*F*_7,48_ = 3.56, *p* < 0.005), but since this was a trivial decrease from 17.6 cycles per minute in the first segment to 14.9 in the last one, these results are not further reported.

**Figure 2:**
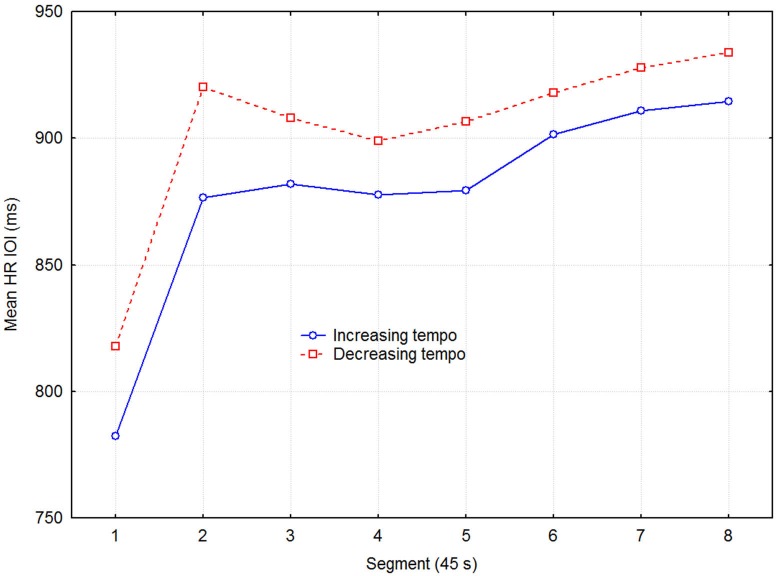
**Mean HR inter-beat-interval as a function of direction of tempo change and time in 45 s segments across the three sessions with the same condition**.

Figure 2 shows the mean HR across all participants, averaged across the three sessions for the same stimulus condition. Heart rate is expressed in terms of the IOI during the recovery phase, separated into eight 45 s segments. Notice the steep increase in IOI over the first two segments where most of the physiological recovery occurred.

**Figure 3:**
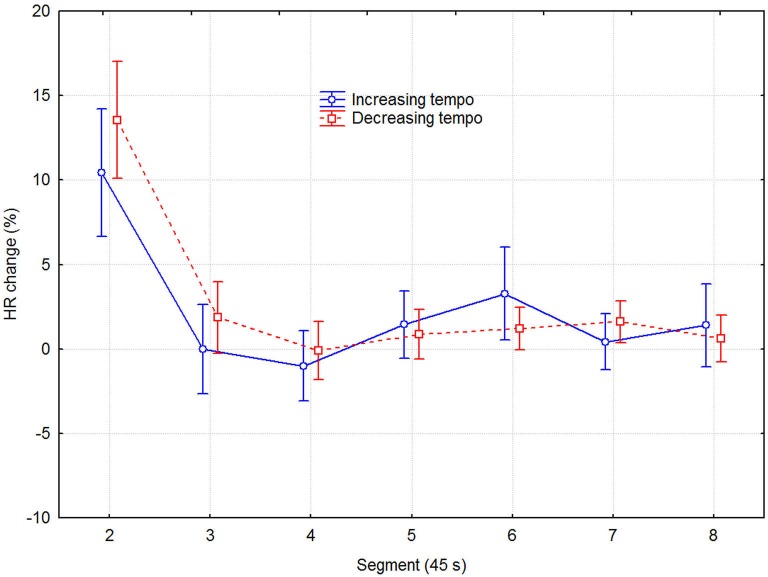
**Mean HR decrease as a function of direction of tempo change and time in 45 s segments**. The values are the differences in percent from the preceding time segment. Error bars indicate 0.95% confidence intervals.

Although separate plots for sessions with increasing and decreasing tempo show that HR was consistently slower when tempo decreased, this difference was not statistically significant, according to a three-way (2 direction of tempo change × 8 recovery time segment × 6 session) repeated measures ANOVA with HR as dependent variable. Only the main effect of recovery time segment was statistically significant (*F*_7,48_ = 12.13, *p* < 0.0001), while those of direction of tempo change (*F*_1,48_ = 3.93, *p* = 0.075) and sessions (*F*_2,48_ = 2.25, *p* = 0.131) were not. No interactions were significant, which together with the lack of main effect of sessions indicated that no learning/ordering effects occurred. That the main effect of tempo change was non-significant, although it was in the expected direction and consistent across both recovery time segment and session, seems to be due to the large variability among participants and across sessions. Effect sizes were small but nevertheless consistent: the grand mean effect size of tempo change was ~0.10, whereas it was 0.24, 0.19, and 0.11 for the first three time segments, respectively. A more detailed examination showed that the means for the tempo conditions were sometimes in the opposite direction to the expected. This was the case for 3 out of the 11 participants for time segment 1, for two other participants for time segment 2, and for another two participants across all eight time segments, for example. To avoid the lack of power of the factorial analysis, we applied a univariate dependent *t*-test across participants, sessions, and time segments, which also showed the effect of tempo change on HR to be just short of significance (*t*_1,526_ = 1.848, *p* = 0.065). Another relevant measure is the proportional change in HR. It was on the order of 2–4% of the mean HR, which is not trivial in this context (Chuang et al., 2010; Eliakim et al., 2013). Figure 3 describes the change in HR during the recovery phase in terms of the proportional decrease of the HR IOI across adjacent time segments. It shows that the initially fast decline in HR levels off somewhere between 135–180 s (segments 3–4).

**Figure 4:**
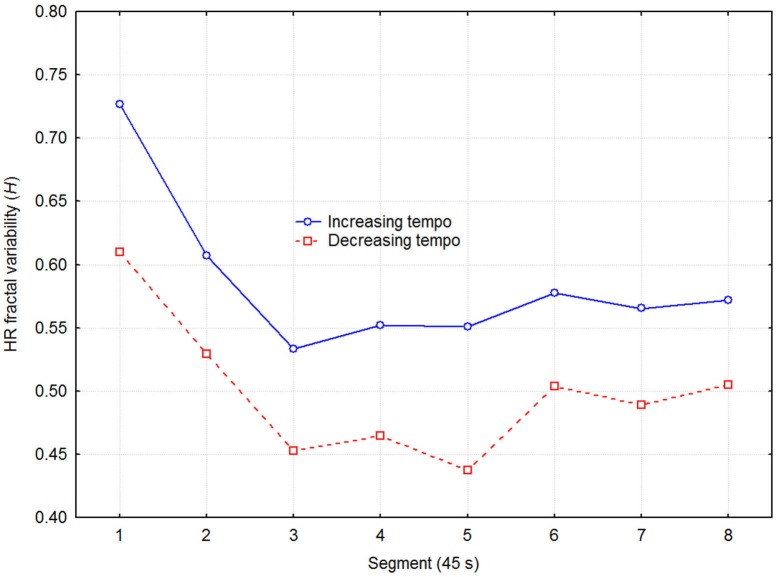
**Fractal dimension of HRV as a function of direction of tempo change and recovery time in 45 s segments, expressed as the Hurst exponent (*H*) estimated with Detrended Fluctuation Analysis (DFA)**. A higher *H* reflects stronger LF power relative to the HF power in the HRV spectrum.

Next, we tested if the trend for lower heart rate when listening to decreasing tempo was related to ANS activity. DFA was used to estimate the LF/HF frequency ratio in terms of the HRV fractal dimension. The DFA produces an estimate of the Euclidian dimension (*D*), which was transformed to the Hurst exponent (*H* = 2-*D*). *H* can assume values from 0 to 1, where 0.5 corresponds to white noise, and 1.0 to very strong positive serial correlation (Brownian motion). A healthy heart operates in the 1/f range between these values, and Figure 4 depicts the present values as a function of direction of tempo change and recovery time segment. A three-way (2 direction of tempo change × 8 recovery time segment × 6 session) ANOVA with *H* as dependent variable indicated significant effects of direction of tempo change (*F*_1,48_ = 6.15, *p* = 0.032), and recovery time segment (*F*_7,48_ = 3.87, *p* = 0.001), while the effect of session and all higher-order interactions were non-significant. Thus, decreasing tempo resulted in relatively weaker low frequency than high frequency variability in participants’ HRV. This suggests, according to the literature, that decreasing tempo caused relatively less sympathetic arousal or more parasympathetic arousal. Again, no effect of sessions on target variable (*H*) was present, showing that no significant effect of any confounding learning/ordering effects was present.

Finally, the direction of the sound pattern had no effects on the relaxation rating (DCR) or the emotion ratings (PANAS), according to one-way ANOVAs.

## Discussion

Decreasing tempo resulted in a comparatively smaller LF/HF component ratio of HRV, which in turn was related to a non-significant trend for lower HR. This is consistent with a host of data indicating that relatively more HF than LF activity reflects relatively more parasympathetic and/or less sympathetic activity in sympathovagal modulation of heart functioning (Pagani et al., 1986, 1997, 2012; Camm et al., 1996; Perini and Veicsteinas, 2003; Chuang et al., 2010; Boudreau et al., 2012). In line with previous research (Camm et al., 1996; Ellis and Thayer, 2010), a probable explanation for the present findings is that the parasympathetic control on heart functioning induced by neuropsychological entrainment to decreasing tempo created an increase in the parasympathetic control of HRV. This emerged as a smaller LF/HF component ratio suggesting enhanced relaxation in this group of physically active adults as they recovered from short, aerobic exercise. During increasing tempo, in contrast, entrainment probably had an arousing effect that induced a larger LF/HF component ratio of HRV, reflecting disordered relaxation.

The study design precluded any direct measurement of entrainment, and we therefore relate how it was deduced to occur. The most obvious evidence is the effect of tempo direction, because this manipulation is extremely unlikely to have any effect unless through entrainment. This is because the local properties of the MLP are almost identical regardless of its direction, and perception of the direction of the tempo change relies on entrainment. The only alternative explanation for the effect would be that the relative duration of adjacent time intervals in the pattern were perceived as such. These differences are likely below the detection threshold, however. The reason one cannot be sure is that being a multiple-level pattern, it is unclear which level of comparison would be used by the operator, and hence which threshold that would correspond to the uni-level patterns employed in previous studies of interval difference detection. Furthermore, previous experiments with the MLP showed ubiquitous and highly accurate entrainment with hand movements, so that this would not take place is extremely far-fetched, also given human’s strong propensity for entrainment and poor time interval discrimination ability. Most likely, entrainment was induced as shown in several studies how neuronal groupings indirectly or directly are enticed to oscillate in time with the fundamentals, harmonics or subharmonics of external rhythms. This electrochemical neuronal phenomenon occurs regardless of whether stimuli is auditory or visual or if the entrainment is voluntary or involuntary (Lakatos et al., 2005, 2008; Gomez-Ramirez et al., 2011; Henry and Obleser, 2012).

The present findings are conceivably generalizable to a wide array of situations, tasks, and other conditions. Since human entrainment consists of what may be characterized as biological reflexes to a considerable extent, it is reasonable to assume that the results derived from this sample of individuals are generalizable to other humans. Across several types, lengths and intensities of exercise, the response in the human body is substantially similar, e.g., the initially increasing HR, plateauing HR and stroke volume after about 2 min, deeper and faster BC, faster PNS withdrawal followed by slower, gradually increasing SNS-activation (Plowman and Smith, 2011). To a considerable extent, this upwards regulatory pattern reverses in a similar fashion during recovery from different exercise protocols, e.g., in decreasing or diminishing HR, heart stroke volume, BC and breathing volume, body temperature, lowered BP, repair of damaged tissue and so forth. Of course there are substantial recovery-differences between moderate cycling for 6 min and running a marathon, but these are also to a substantial degree dose-response related and qualitatively similar. This supports substantial generalization of the findings to recovery from other exercise conditions although precise limits for other recovery-responses need to be investigated with high experimental control similar to this study. The stimulus characteristics are also deterministic, replicable, and have well-defined higher-order properties that go beyond this particular implementation of the perceptual constructs “increasing tempo” and “decreasing tempo”. This means that strong conclusions can be drawn that the differences in the dependent variable is caused by the perceived tempo direction of the stimulus. Because the stimulus properties were pure in the sense that both conditions were locally identical in terms of pitch, event density and so forth, and differed only in the higher-order property “direction of tempo change” it is highly probable that the small but significant physiological change was caused by, and only by, rhythmic entrainment.

Future research should replicate the present findings, including larger samples, since there was a non-significant tendency (*p* = 0.075) in the predicted direction of the main hypothesis of faster relaxation reflected by mean HR (Figure 2). If allowed to speculate on non-significant results, this seems to harmonize well with other findings that indicate an effect of rhythmic stimuli on HRV but not on HR (Ellis, 2009). One should however consider that those studies used isochronous rhythmic stimuli, which carry less temporal information than does the MLP, and which also do not change tempo. The effects of the MLP are largely uncharted but can be expected to be more potent for entrainment purposes (Madison and Merker, 2005) due to richer information or higher event density. We also failed to find any effect of tempo change direction on pulmonary activity, and conclude that at least in this particular experimental setting, BC is not a valid measure of entrainment. One limitation of BC is that it only considers number of cycles in relation to time. Consequently, BC ignores depth of the breathing cycles. It was qualitatively noted that participants seemed to start taking heavier breaths shortly after exercise was initiated, a pattern that seemed to reverse during recovery as participants returned to lighter breathing. By also considering total breathing volume one may improve the study of entrainment in relation to breathing.

The effect found for the direction of tempo change on HRV proves with almost certainty that participants in this study did indeed entrain to the stimulus in the expected direction, although, as mentioned in the Introduction section, their relaxation and recovery precluded any form of overt behavior. The presence of physiological responses validates further use of this new kind of stimulus to study entrainment phenomena. A stimulus that slowly changes its local pulse systematically may also shed particular light on ritardando and accelerando aspects of music, such as the sometimes gradually decreasing tempo at the end of a ballad or increasing tempo of a piece of Russian folk music, which are ecologically valid, entrainable phenomena closely similar to the stimulus used. Ritardando and accelerando are particularly demanding of timing capabilities in musicians, used as expressive features and not extensively studied (Schultze et al., 2005).

There were no effects on ratings of relaxation or affect, although the PANAS is considered to be quite sensitive (Crawford and Henry, 2004). Given that physiological effects were found, it seems they were too small to be consciously detected. This explanation suggests that this kind of intervention exerts its influence on physiological functioning directly rather than indirectly by altering how participants perceive their exertion or motivation. The MLP incorporates a central musical feature; rhythm. However, in this study it was also bereft of further complexity so common in music and its effect was physiological but not psychological. This may align with a possible pattern of more erratic physiological and more robust psychological results found on target variables when stimulus complexity increases as more music-like stimuli are used. Also, the opposite seems reasonable when stimulus is stripped of complexity and much of its musical qualities. For such highly controlled stimuli only in part reminiscent of music, physiological effects are perhaps more law-bound and consistent but because most musical features are removed, psychological effects tend to diminish and thusly generate inconsistency. Because the MLP is different from musical constructs and no effects on psychological variables was found, it indirectly points to the idea that music’s inherent complexity affects us by means of our psychological subjectivity rather than our biological reflexes. This may improve our understanding of why physiological effects of music are inconclusive (Bartlett, 1996) and yet we persistently and subjectively advocate its central effect on us (Priest and Karageorghis, 2008).

Although the synthetic nature of the MLP stimulus may confer limits in terms of ecological validity, it can be adopted to different musical structures, adding more layers such as harmonic and melodic movement, increasing its resemblance to real music. With such alterations, it could within the artificial limits of the MLP be possible to disentangle the effects on music style, harmony, and melody from those of tempo change, since the stimulus is otherwise identical.

Some limitations of the present study should be discussed. The design does not allow comparisons of the MLP with silence or other non-MLP conditions, since this study focused on the effect of the tempo change direction. Although it caused a significant difference in HR variability, we cannot tell whether the relative difference in the LF/HF component ratio of HRV reflects enhanced relaxation during decreasing tempo or an induced stress response during increasing tempo. Hence, a future study aimed at investigating absolute differences between conditions compared to a control condition of an isochronous version of the MLP holds potential for furthering our understanding of this aspect.

Considering implications in the world of sports and exercise, particularly between short bouts of training or performance as suggested initially, physically active adults are thought to have limited use of this method because the effect size was small. Indeed, probably smaller than those of other methods to enhance recovery. On the other hand, the use of this method to aid recovery more indirectly or preventively may yield larger benefits, as if used when having longer periods of recovery. A recent study found that the effect of a rhythmic stimulus on recovery was largest at the end of the recovery period (Eliakim et al., 2013) and a fairly recent review on randomized control trial studies showed that recovery time increases the recovering response independent of recovery-type (Pastre et al., 2009).

As most of this research is still in its infancy, possible future progression yields promise for beneficial applications. The experimental effect of the MLP-induced, rhythmic entrainment on LF/HF of HRV during recovery from aerobic exercise was expectedly small, yet also causally strong. Many human activities are rhythmic in different ways (Kolb and Wishaw, 2005; Ellis and Thayer, 2010), such as turn-taking in spoken dialog or in precise time-locking in joint musical behavior, dance and drill (Greenfield, 1994; McNeill, 1995; Merker et al., 2009). One could speculate that tempo change, because of its possibility for entrainment and coupled physiological effect, may in some ways be superior to other methods to recover after exercise. Future research on the MLP may therefore lead to the development of practical interventions for individuals who need to enhance recovery after exercise due to problematic conditions, perhaps if they train or perform in close proximity to their sleep-period or if they suffer from anxiety or stress. Since potential side-effects are mild and the monetary costs low for sound patterns compared to psychotropic drugs, the possibility to create practical applications using the entrainment phenomenon to the benefit of human physiological functioning merits further scientific exploration.

## Author contributions

John Wallert and Guy Madison conceived and designed the experiment. John Wallert and Guy Madison performed the experiment. John Wallert and Guy Madison analyzed the data. Guy Madison contributed reagents/materials/analysis tools. John Wallert and Guy Madison wrote the paper.

## Conflict of interest statement

The authors declare that the research was conducted in the absence of any commercial or financial relationships that could be construed as a potential conflict of interest.

## References

[B1] Akselrod S. Gordon D. Ubel F. A. Shannon D. C. Barger A. C. Cohen J. R. (1981). Power spectrum analysis of heart rate fluctuation: a quantitative probe of beat-to-beat cardiovascular control. Science 213, 220–222 10.1126/science.6166045 6166045

[B2] Andersson G. (2011). Nya Konditionstest På Cykel. Stockholm: SISU Idrottsböcker

[B3] Åstrand P.-O. Rodahl K. Dahl H. A. Strømme S. B. (2003). Textbook of Work Physiology: Physiological Bases of Exercise. Champaign, IL: Human Kinetics

[B4] Barker L. W. (1991). “The use of music and relaxation techniques to reduce pain of burn patients during daily debridement,” in Applied Music Medicine, ed Maranto C. (Washington, DC: National Association for Music Therapy), 124–140

[B5] Bartlett D. L. (1996). “Physiological responses to music and sound stimuli,” in Handbook of Music Psychology, ed Hodges D. A. (San Antonio: IMR Press), 343–385

[B6] Birmingham C. L. Tan A. O. (2003). Respiratory muscle weakness and anorexia nervosa. Int. J. Eat. Disord. 33, 230–233 10.1002/eat.10131 12616590

[B7] Bollaert P. E. Gimenez M. Robinherbier B. Escanye J. M. Mallie J. P. Robert J. (1992). Respective effects of malnutrition and phosphate-depletion on endurance swimming and muscle metabolism in rats. Acta Physiol. Scand. 144, 1–7 10.1111/j.1748-1716.1992.tb09260.x 1595346

[B8] Boudreau P. Yeh W. H. Dumont G. A. Boivin D. B. (2012). A circadian rhythm in heart rate variability contributes to the increased cardiac sympathovagal response to awakening in the morning. Chronobiol. Int. 29, 757–768 10.3109/07420528.2012.674592 22734576

[B9] Budgett R. (1998). Fatigue and underperformance in athletes: the overtraining syndrome. Br. J. Sports Med. 32, 107–110 10.1136/bjsm.32.2.107 9631215 PMC1756078

[B10] Camm A. J. Malik M. Bigger J. T. Breithardt G. Cerutti S. Cohen R. J. (1996). Heart rate variability. Standards of measurement, physiological interpretation and clinical use. Eur. Heart J. 17, 354–381 10.1093/oxfordjournals.eurheartj.a014868 8737210

[B11] Cantor D. S. Stevens E. (2009). QEEG correlates of auditory-visual entrainment treatment efficacy of refractory depression. J. Neurother. 13, 100–108 10.1080/10874200902887130

[B12] Cepeda M. S. Carr D. B. Lau J. Alvarez H. (2006). Music for Pain Relief [Online]. Issue 2. Available online at: http://onlinelibrary.wiley.com/doi/10.1002/14651858.CD004843.pub2/full Accessed on 19 February 2014.

[B13] Chuang C. Y. Han W. R. Li P. C. Young S. T. (2010). Effects of music therapy on subjective sensations and heart rate variability in treated cancer survivors: a pilot study. Complement. Ther. Med. 18, 224–226 10.1016/j.ctim.2010.08.003 21056846

[B14] Clayton M. (2012). What is entrainment? Definition and applications in musical research. Empir. Musicol. Rev. 7, 49–56

[B15] Conley K. M. Lehman B. J. (2012). Test anxiety and cardiovascular responses to daily academic stressors. Stress Health 28, 41–50 10.1002/smi.1399 22259157

[B16] Crawford J. R. Henry J. D. (2004). The positive and negative affect schedule (PANAS): construct validity, measurement properties and normative data in a large non-clinical sample. Br. J. Clin. Psychol. 43, 245–265 10.1348/0144665031752934 15333231

[B17] Demello J. J. Cureton K. J. Boineau R. E. Singh M. M. (1987). Ratings of percieved exertion at the lactate threshold in trained and untrained men and women. Med. Sci. Sports Exerc. 19, 354–362 10.1249/00005768-198708000-00006 3657484

[B18] de Niet G. Tiemens B. Lendemeijer B. Hutschemaekers G. (2009). Music-assisted relaxation to improve sleep quality: meta-analysis. J. Adv. Nurs. 65, 1356–1364 10.1111/j.1365-2648.2009.04982.x 19456998

[B19] Eliakim M. Bodner E. Meckel Y. Nemet D. Eliakim A. (2013). Effect of rhythm on the recovery from intense exercise. J. Strength Cond. Res. 27, 1019–1024 10.1519/jsc.0b013e318260b829 22692126

[B20] Ellis R. J. (2009). “The effect of musical tempo on subjective and physiological indices of affective response”. Doctoral Dissertation (Ohio State University).

[B21] Ellis R. J. Thayer J. F. (2010). Music and autonomic nervous system (Dys)function. Music Percept. 27, 317–326 10.1525/mp.2010.27.4.317 21197136 PMC3011183

[B22] Garcia D. Moradi S. (2012). The affective temperaments and well-being: Swedish and Iranian adolescents’ life satisfaction and psychological well-being. J. Happiness Stud. 14, 689–707 10.1007/s10902-012-9349-z

[B23] Gilenstam K. M. Thorsen K. Henriksson-Larsén K. B. (2011). Physiological correlates of skating performance in women’s and men’s ice hockey. J. Strength Cond. Res. 25, 2133–2142 10.1519/jsc.0b013e3181ecd072 21785292

[B24] Gomez-Ramirez M. Kelly S. P. Molholm S. Sehatpour P. Schwartz T. H. Foxe J. J. (2011). Oscillatory sensory selection mechanisms during intersensory attention to rhythmic auditory and visual inputs: a human electrocorticographic investigation. J. Neurosci. 31, 18556–18567 10.1523/jneurosci.2164-11.2011 22171054 PMC3298747

[B25] Good M. (1995). A comparison of the effects of jaw relaxation and music on postoperative pain. Nurs. Res. 44, 52–57 10.1097/00006199-199501000-00010 7862546

[B26] Greenfield M. D. (1994). Cooperation and conflict in the evolution of signal interactions. Annu. Rev. Ecol. Syst. 25, 97–126 10.1146/annurev.ecolsys.25.1.97

[B27] Guzzetta C. E. (1989). Effects of relaxation and music therapy on patients in a coronary care unit with presumptive acute myocardial infarction. Heart Lung 18, 609–616 2684920

[B28] Halsband U. Mueller S. Hinterberger T. Strickner S. (2009). Plasticity changes in the brain in hypnosis and meditation. Contemp. Hypn. 26, 194–215 10.1002/ch.386

[B29] Heffernan K. S. Fahs C. A. Shinsako K. K. Jae S. Y. Fernhall B. (2007). Heart rate recovery and heart rate complexity following resistance exercise training and detraining in young men. Am. J. Physiol. Heart Circ. Physiol. 293, H3180–H3186 10.1152/ajpheart.00648.2007 17890428

[B30] Heinrichs-Graham E. Wilson T. W. (2012). Presence of strong harmonics during visual entrainment: a magnetoencephalography study. Biol. Psychol. 91, 59–64 10.1016/j.biopsycho.2012.04.008 22569101 PMC3407313

[B31] Henry M. J. Obleser J. (2012). Frequency modulation entrains slow neural oscillations and optimizes human listening behavior. Proc. Natl. Acad. Sci. U S A 109, 20095–20100 10.1073/pnas.1213390109 23151506 PMC3523826

[B32] Higginbotham M. B. Morris K. G. Williams R. S. McHale P. A. Coleman R. E. Cobb F. R. (1986). Regulation of stroke volume during submaximal and maximal upright exercise in normal man. Circ. Res. 58, 281–291 10.1161/01.res.58.2.281 3948345

[B33] Imai K. Sato H. Hori M. Kusuoka H. Ozaki H. Yokoyama H. (1994). Vagally mediated heart rate recovery after exercise is accelerated in athletes but blunted in patients with chronic heart failure. J. Am. Coll. Cardiol. 24, 1529–1535 10.1016/0735-1097(94)90150-3 7930286

[B34] Iwanaga M. Kobayashi A. Kawasaki C. (2005). Heart rate variability with repetitive exposure to music. Biol. Psychol. 70, 61–66 10.1016/j.biopsycho.2004.11.015 16038775

[B35] Iwanaga M. Moroki Y. (1999). Subjective and physiological responses to music stimuli controlled over activity and preference. J. Music Ther. 36, 26–38 10.1093/jmt/36.1.26 10519843

[B36] Johansson A. M. Domellöf E. Rönnqvist L. (2012). Short- and long-term effects of synchronized metronome training in children with hemiplegic cerebral palsy: a two case study. Dev. Neurorehabil. 15, 160–169 10.3109/17518423.2011.635608 22296344

[B37] Jones M. R. Moynihan H. MacKenzie N. Puente J. (2002). Temporal aspects of stimulus driven attending in dynamic arrays. Psychol. Sci. 13, 313–319 10.1111/1467-9280.00458 12137133

[B38] Karageorghis C. I. Terry P. C. (2008). “The psychological, psychophysical and ergogenic effects of music in sport: a review and synthesis,” in Sporting Sounds: Relationships Between Sport and Music, ed Bateman A. J. Bale J. R. (London: Taylor Francis), 14–36

[B39] Kolb B. Wishaw I. Q. (2005). An Introduction to Brain and Behavior. New York: Worth Publishers

[B40] Kontsas K. Triantafyllidi H. Trivilou P. Ikonomidis I. Tzortzis S. Liazos I. (2013). Delayed blood pressure recovery ratio might indicate increased arterial stiffness in hypertensive patients with reduced aerobic exercise capacity. Blood Press. 22, 290–296 10.3109/08037051.2012.759694 23373532

[B41] Lakatos P. Karmos G. Mehta A. D. Ulbert I. Schroeder C. E. (2008). Entrainment of neuronal oscillations as a mechanism of attentional selection. Science 320, 110–113 10.1126/science.1154735 18388295

[B42] Lakatos P. Shah A. S. Knuth K. H. Ulbert I. Karmos G. Schroeder C. E. (2005). An oscillatory hierarchy controlling neuronal excitability and stimulus processing in the auditory cortex. J. Neurophysiol. 94, 1904–1911 10.1152/jn.00263.2005 15901760

[B43] Larsson P. Henriksson-Larsén K. (2005). Combined metabolic gas analyser and dGPS analysis of performance in cross-country skiing. J. Sports Sci. 23, 861–870 10.1080/02640410400022078 16195038

[B44] Lavallee C. F. Koren S. A. Persinger M. A. (2011). A quantitative electroencephalographic study of meditation and binaural beat entrainment. J. Altern. Complement. Med. 17, 351–355 10.1089/acm.2009.0691 21480784

[B45] Lazic S. E. Ogilvie R. D. (2007). Lack of efficacy of music to improve sleep: a polysomnographic and quantitative EEG analysis. Int. J. Psychophysiol. 63, 232–239 10.1016/j.ijpsycho.2006.10.004 17123654

[B46] Loft P. Thomas M. G. Petrie K. J. Booth R. J. Miles J. Vedhara K. (2007). Examination stress results in altered cardiovascular responses to acute challenge and lower cortisol. Psychoneuroendocrinology 32, 367–375 10.1016/j.psyneuen.2007.02.004 17395393

[B47] Lunt H. C. Corbett J. Barwood M. J. Tipton M. J. (2011). Cycling cadence affects heart rate variability. Physiol. Meas. 32, 1133–1145 10.1088/0967-3334/32/8/009 21693796

[B48] Lykken D. Tellegen A. (1996). Happiness is a stochastic phenomenon. Psychol. Sci. 7, 186–189 10.1111/j.1467-9280.1996.tb00355.x

[B49] Madison G. (2004). Fractal modeling of human isochronous serial interval production. Biol. Cybern. 90, 105–112 10.1007/s00422-004-0482-6 14999477

[B50] Madison G. (2006). Duration specificity in the long-range correlation of human serial interval production. Physica D 216, 301–306 10.1016/j.physd.2006.03.002

[B51] Madison G. (2009). An auditory illusion of infinite tempo change based on multiple temporal levels. PLoS One 4:e8151 10.1371/journal.pone.0008151 19997635 PMC2780720

[B52] Madison G. Merker B. (2005). Timing of action during and after synchronization with linearly changing levels. Music Percept. 22, 441–459 10.1525/mp.2005.22.3.441

[B53] McNeill W. H. (1995). Keeping Together in Time: Dance and Drill in Human History. Massachusetts: Harvard University Press

[B54] Merker B. H. Madison G. S. Eckerdal P. (2009). On the role and origin of isochrony in human rhythmic entrainment. Cortex 45, 4–17 10.1016/j.cortex.2008.06.011 19046745

[B55] Mika A. Mika P. Fernhall B. Unnithan V. B. (2007). Comparison of recovery strategies on muscle performance after fatiguing exercise. Am. J. Phys. Med. Rehabil. 86, 474–481 10.1097/phm.0b013e31805b7c79 17515687

[B56] Otsuki T. Maeda S. Iemitsu M. Sait Y. Tanimura Y. Sugawara J. (2007). Postexercise heart rate recovery accelerates in strength-trained athletes. Med. Sci. Sports Exerc. 39, 365–370 10.1249/01.mss.0000241647.13220.4c 17277602

[B57] Pagani M. Lombardi F. Guzzetti S. Rimoldi O. Furlan R. Pizzinelli P. (1986). Power spectral analysis of heart rate and arterial pressure variabilities as a marker of sympatho-vagal interaction in man and conscious dog. Circ. Res. 59, 178–193 10.1161/01.res.59.2.178 2874900

[B58] Pagani M. Lucini D. Porta A. (2012). Sympathovagal balance from heart rate variability: time for a second round? Exp. Physiol. 97, 1141–1142 10.1113/expphysiol.2012.066977 23015734

[B59] Pagani M. Montano N. Porta A. Malliani A. Abboud F. M. Birkett C. (1997). Relationship between spectral components of cardiovascular variabilities and direct measures of muscle sympathetic nerve activity in humans. Circulation 95, 1441–1448 10.1161/01.cir.95.6.1441 9118511

[B60] Pastre C. M. Bastos F. D. Netto J. Vanderlei L. C. M. Hoshi R. A. (2009). Post-exercise recovery methods: a systematic review. Rev. Bras. Med. Esporte 15, 138–144 10.1590/S1517-86922009000200012

[B61] Peng C. K. Buldyrev S. V. Goldberger A. L. Havlin S. Mantegna R. N. Simons M. (1995). Statistical properties of DNA sequences. Physica A 221, 180–192 10.1016/0378-4371(95)00247-5 11540495

[B62] Peng C. K. Buldyrev V. Havlin S. Simons M. Stanley H. E. Goldberger A. L. (1994). Mosaic organization of DNA nucleotides. Phys. Rev. E Stat. Phys. Plasmas Fluids Relat. Interdiscip. Topics 49, 1685–1689 10.1103/physreve.49.1685 9961383

[B63] Perini R. Veicsteinas A. (2003). Heart rate variability and autonomic activity at rest and during exercise in various physiological conditions. Eur. J. Appl. Physiol. 90, 317–325 10.1007/s00421-003-0953-9 13680241

[B64] Phillips-Silver J. Aktipis C. A. Bryant G. A. (2010). The ecology of entrainment: foundations of coordinated rhythmic movement. Music Percept. 28, 3–14 10.1525/mp.2010.28.1.3 21776183 PMC3137907

[B65] Phillips-Silver J. Keller P. E. (2012). Searching for roots of entrainment and joint action in early musical interactions. Front. Hum. Neurosci. 6:26 10.3389/fnhum.2012.00026 22375113 PMC3288575

[B66] Plowman S. A. Smith D. L. (2011). “Cardiovascular response to exercise,” in Exercise Physiology for Health, Fitness and Performance, ed Plowman S. A. (Baltimore, MD: Lippincott Williams Wilkins), 355–387

[B67] Pothoulaki M. Macdonald R. A. Flowers P. Stamataki E. Filiopoulos V. Stamatiadis D. (2008). An investigation of the effects of music on anxiety and pain perception in patients undergoing haemodialysis treatment. J. Health Psychol. 13, 912–920 10.1177/1359105308095065 18809642

[B68] Priest D. L. Karageorghis C. I. (2008). A qualitative investigation into the characteristics and effects of music accompanying exercise. Eur. Phys. Educ. Rev. 14, 347–366 10.1177/1356336x08095670

[B69] Samuels C. (2009). Sleep, recovery and performance: the new frontier in high-performance athletics. Phys. Med. Rehabil. Clin. N. Am. 20, 149–159 10.1016/j.pmr.2008.10.009 19084768

[B70] Sayers B. M. (1973). Analysis of heart rate variability. Ergonomics 16, 17–32 10.1080/0014013730892447 4702060

[B71] Schultze H.-H. Cordes A. Vorberg D. (2005). Keeping synchrony while tempo changes: Accelerando and Ritardando. Music Percept. 22, 461–477 10.1525/mp.2005.22.3.461

[B72] Seiler S. Haugen O. Kuffel E. (2007). Autonomic recovery after exercise in trained athletes: intensity and duration effects. Med. Sci. Sports Exerc. 39, 1366–1373 10.1249/mss.0b013e318060f17d 17762370

[B73] Shvartz E. Reibold R. C. (1990). Aerobic norms for males and females aged 6 to 75 years: a review. Aviat. Space Environ. Med. 61, 3–11 2405832

[B74] Sinacore D. R. Coyle E. F. Hagberg J. M. Holloszy J. O. (1993). Histochemical and physiological correlates of training- and detraining-induced changes in the recovery from a fatigue test. Phys. Ther. 73, 661–667 8378422 10.1093/ptj/73.10.661

[B75] Smith J. C. Joyce C. A. (2004). Mozart versus new age music: relaxation states, stress and ABC relaxation theory. J. Music Ther. 41, 215–224 10.1093/jmt/41.3.215 15327344

[B76] Sommer M. Rönnqvist L. (2009). Improved motor-timing: effects of synchronized metronome training on golf shot accuracy. J. Sports Sci. Med. 8, 648–656 24149608 PMC3761554

[B77] Stefanics G. Hangya B. Hernádi I. Winkler I. Lakatos P. Ulbert I. (2010). Phase entrainment of human delta oscillations can mediate the effects of expectation on reaction speed. J. Neurosci. 30, 13578–13585 10.1523/jneurosci.0703-10.2010 20943899 PMC4427664

[B78] Thaut M. H. McIntosh G. C. Rice R. R. (1997). Rhythmic facilitation of gait training in hemiparetic stroke rehabilitation. J. Neurol. Sci. 151, 207–212 10.1016/S0022-510X(97)00146-9 9349677

[B79] Thayer J. F. Lane R. D. (2000). A model of neurovisceral integration in emotion regulation and dysregulation. J. Affect. Disord. 61, 201–216 10.1016/s0165-0327(00)00338-4 11163422

[B80] Thayer J. F. Lane R. D. (2009). Claude Bernard and the heart-brain connection: further elaboration of a model of neurovisceral integration. Neurosci. Biobehav. Rev. 33, 81–88 10.1016/j.neubiorev.2008.08.004 18771686

[B81] Tomlin D. L. Wenger H. A. (2001). The relationship between aerobic fitness and recovery from high intensity intermittent exercise. Sports Med. 31, 1–11 10.2165/00007256-200131010-00001 11219498

[B82] Uijtdehaage S. H. J. Thayer J. F. (2000). Accentuated antagonism in the control of the human heart. Clin. Auton. Res. 10, 107–110 10.1007/bf02278013 10954067

[B83] Vasionytė I. Madison G. (2013). Musical intervention for patients with dementia: a meta-analysis. J. Clin. Nurs. 22, 1203–1216 10.1111/jocn.12166 23574287

[B84] Ward L. M. (2003). Synchronous neural oscillations and cognitive processes. Trends Cogn. Sci. 7, 553–559 10.1016/j.tics.2003.10.012 14643372

[B85] Watson D. Clark L. A. Tellegen A. (1988). Development and validation of brief measures of positive and negative affect: the PANAS scales. J. Pers. Soc. Psychol. 54, 1063–1070 10.1037/0022-3514.54.6.1063 3397865

[B86] Weippert M. Behrens K. Rieger A. Stoll R. Kreuzfeld S. (2013). Heart rate variability and blood pressure during dynamic and static exercise at similar heart rate levels. PLoS One 8:e83690 10.1371/journal.pone.0083690 24349546 PMC3862773

[B87] Williams J. M. Harris D. V. (2006). Applied Sport Psychology: Personal Growth to Peak Performance. NY: McGraw-Hill

[B88] Yamashita S. Iwai K. Akimoto T. Sugawara J. Kono I. (2006). Effects of music during exercise on RPE, heart rate and the autonomic nervous system. J. Sports Med. Phys. Fitness 46, 425–430 16998447

[B89] Yu X. Fumoto M. Nakatani Y. Sekiyama T. Kikuchi H. Seki Y. (2011). Activation of the anterior prefrontal cortex and serotonergic system is associated with improvements in mood and EEG changes induced by Zen meditation practice in novices. Int. J. Psychophysiol. 80, 103–111 10.1016/j.ijpsycho.2011.02.004 21333699

[B90] Zhou B. Conlee R. K. Jensen R. Fellingham G. W. George J. D. Fisher A. G. (2001). Stroke volume does not plateau during graded exercise in elite male distance runners. Med. Sci. Sports Exerc. 33, 1849–1854 10.1097/00005768-200111000-00008 11689734

